# Prevalence of Cattle Trypanosomosis and Apparent Density of Its Fly Vectors in Bambasi District of Benishangul-Gumuz Regional State, Western Ethiopia

**DOI:** 10.1155/2020/8894188

**Published:** 2020-09-15

**Authors:** Morka Amante, Hika Tesgera

**Affiliations:** ^1^School of Veterinary Medicine, Wollega University, P.O. Box 395, Nekemte, Ethiopia; ^2^Bedelle National Tsetse and Trypanomososis Investigation and Control Center, Bedele, Ethiopia

## Abstract

Trypanosomosis is the most serious disease of cattle, which causes great socioeconomic losses in the country. Its socioeconomic impact is reflected on direct losses due to mortality, morbidity, and reduction in milk and meat production, abortion and stillbirth, and also costs associated with combat of the disease are direct losses. A cross-sectional study was carried out to assess the prevalence of cattle trypanosomosis, and the apparent density and distribution of its fly vectors in selected study areas. The methods employed during the study were buffy coat technique for parasitological study and deploying trap for the collection of tsetse flies. A total of 1512 flies were trapped, and among them, 1162 were tsetse flies while 350 were biting flies. Higher apparent density for tsetse fly (7.7 F/T/D) followed by *Stomoxys* (0.9 F/T/D), *Tabanus* (0.8 F/T/D), and *Hematopota* (0.6 F/T/D) was recorded. Out of 638 examined cattle, the overall prevalence of trypanosomosis in the study area was 9.1% (58/638). Out of positive cases, *Trypanosoma congolense* (7.7%) was the dominant trypanosome species followed by *Trypanosoma vivax* (0.9%), *Trypanosoma brucei* (0.2%), and mixed infection of *Trypanosoma brucei* and *Trypanosoma vivax* (0.3%)^.^ There was no a significant difference (*p* > 0.05) in trypanosome infection between age, sex, and trypanosome species. The prevalence of trypanosomosis on the bases of body condition was 2.8% for poor, 5.5% for medium, and 0.8% for good body condition. The overall prevalence of anemia was (36.8%), and presence of anemia was higher in trypanosome positive animals (62.5%) than in negative animals (34.3%) which is statistically significant (*p* < 0.05, CI = 1.794–5.471). The overall mean packed cell volume (PCV) value for examined animals was 25.84 ± 0.252SE. Mean (PCV) of parasitaemic cattle (9.1%) was significantly (*p* < 0.05) lower than that of aparasitaemic cattle (90%). This survey showed that trypanosomosis is still a core problem for livestock production of the study area. Therefore, more attention should be given to the control of both the disease and its vectors.

## 1. Introduction

Ethiopia has a huge and diverse livestock population that plays an important role in the economies and livelihoods of farmers and pastoralists. Livestock are a “living bank” or “living account” for rural and urban poor farmers or livestock owners. They serve as a financial reserve for the period of economic distress such as crop failure as well as primary cash income. Among livestock, cattle are the primary resource for people in Ethiopia. Despite the large animal population, productivity in Ethiopia is low and even below the average for most countries in Eastern and Sub-Saharan African countries, due to poor nutrition, reproduction problems, management constraints, and prevailing animal disease [1].

In fact, livestock may be affected by a variety of diseases and a number of other unhealthy circumstances. Among these, trypanosomosis is one of the major animal health constraints to livestock production in Sub-Saharan Africa [2]. Bovine trypanosomosis is one of the major impediments to livestock development and agricultural production in Ethiopia contributing negatively to the overall development in general and to food self-reliance efforts of the nation in particular [3].

Trypanosomosis is a protozoan disease of both human and animals caused by different species of the genus *Trypanosoma*, unicellular parasites found in the blood and other tissues of vertebrates include livestock, wild life, and people. It is the one of the most important livestock diseases in Sub-Saharan Africa (SSA) and is present in 37 countries in that region. Trypanosomosis is transmitted to man and animals by a blood-sucking insect, the tsetse fly. Trypanosomosis limited the extension of natural herds, particularly in Africa, where exists the presence of the tsetse fly density access to woodland and savanna areas with good grazing potential [4].

*Trypanosoma* infects cattle, sheep, camels, goats, horses, and many other domestic and wild mammals. The *Trypanosoma* species pathogenic to cattle and known to exist in Ethiopia are *T. congolense*, *T. vivax*, and *T. brucei*. They are distributed mainly in the tsetse belt (where the tsetse fly vector exists) of the country, West, Southwest, and southern part of Ethiopia. *T. vivax* is also found in areas outside of the tsetse belt, where it can possibly be transmitted by mechanical vectors as biting flies [3]. The prevalence varies from locality to locality depending on agroclimatic conditions, seasons, and as part of activities which are intended to control the impact of the disease [5].

Hence, trypanosomosis is a very serious disease of cattle, which causes great socioeconomic losses in the country. Its socioeconomic impact is reflected on direct losses due to mortality, morbidity, and reduction in milk and meat production, abortion and stillbirth, and also costs associated with combat of the disease are direct losses. And hence, studying the prevalence and magnitude of the vector is inevitably important to develop appropriate control measures [3].

In Ethiopia, as mentioned, the disease is more prevalent in the southern and western regions where the primary vector exists. However, in Bambasi District, trypanosomosis was found to be one of the factors that hampered livestock rearing in most peasant associations from researcher observation. Thus, a study on the status of the disease investigating the vectors and their relative abundance is crucial for a successful control in the area. The knowledge of the status of the disease prevalence, its health impact on animals affected, its vector distribution, and the associated risks are very important for understanding the epidemiology of the disease and to devise suitable control measures.

Therefore, the objectives of the study were as follows:

- To determine the prevalence of cattle trypanosomosis found in selected areas of Bambasi District (Northwest Ethiopia)
- To determine apparent densities, distribution, and species of tsetse flies and other biting flies in the selected areas

## 2. Materials and Methods

### 2.1. Study Area

The study was conducted from November 2016 to March 2017 in six peasant associations (PAs) of Bambasi District of Assosa Zone, Benishangul-Gumuz Regional State. Bambasi District has 38 kebeles (PAs), stretches over an area of 2210.16 (km^2^) with a human population of 62693.The region is found in the northwest of the country between the latitude of 9 and 11°N and longitude of 34 and 35°E, and its altitude range is 1500–1900 meter above sea level. Annual rain fall is between 1350–1400 mm with unimodal type of rain fall that occurs between April and October. The annual temperature ranges between 21°C–35°C. The livelihood of the society largely depends on mixed livestock and crop production having a livestock population of 36,735 cattle, 10732 goat, 3739 sheep, 4467 equines, 41438 poultry, and 23423 beehives [6].

### 2.2. Study Animals and Sample Size

The study was conducted on indigenous cattle breed kept under the extensive husbandry management system from six peasant associations (PAs). The sample size required for assessing the prevalence of trypanosomosis in this study was determined using the formula given by [7] for multisample stage sampling, by using 95% level of confidence interval, 50% expected prevalence, and 0.05 desired absolute precision. Thus, 384 cattle were needed for the study. However, to increase the precision, 638 were included.

### 2.3. Study Design

The PAs were selected based on their accessibility to transport and information from the district's administrative body. Multistage sampling was used to sample animals, where herds were selected from each PA by simple random sampling as a primary sampling unit. From selected herds, individual animals to be sampled were selected by simple random sampling technique as secondary sampling unit.

The age of sampled animals was determined based on owners' information, and they were grouped as young (≤2 years of age), adults (>2 ≤ 5 years), and old (>5 years). Body condition of animals was determined by physical appearance and observation of body conformation.

### 2.4. Study Methodology

#### 2.4.1. Entomological Survey

An entomological study was also carried out to assess the apparent density and the species of tsetse and other biting flies in the study area. During the study, 75 traps were deployed in five selected sites which seemed suitable habitat for tsetse flies. The coordinates of each trap position were recorded with a Global Positioning System (GPS). A total of 55 monoconical and 20 bioconical traps were deployed in five PAs of the district at approximate intervals of 200–250 m. All traps were baited with acetone, octenol, and cow urine filled in separate bottles. After 48 hours of deployment, tsetse flies in the cages were counted and identified based on their habitat (the palpalis group found around lakes and river, fusca groups found in dense forest, and the morsitans group found in savannah area) and morphology to the genus and species level. Other biting flies were also identified according to their morphological structures, such as size and proboscis at the genus level.

#### 2.4.2. Survey on Trypanosomosis

Blood samples were collected randomly from cattle of the settlement into the capillary tube after piercing the ear vein by using a lancet. One end of the capillary tube was sealed and centrifuged at 12,000 rpm for 5 minutes to separate the blood cells and to concentrate trypanosomes using centrifugal forces to form the buffy coat. Then, the mean PCV was measured using a hematocrit reader. The capillary tubes were broken just 1 mm below the buffy coat and expressed on microscopic slide, mixed and covered with 22 × 22 mm cover slip. Then, the slide was examined under a 40x objective of a compound light microscope using the dark ground buffy coat technique to detect the presence of the parasite. For positive cases, in Giemsa-stained blood smears, the morphology of the species can be distinguished by their size, shape, location and size of kinatoplast, position of nucleus, and the attachment and length of flagellum.

### 2.5. Data Analysis

All the collected raw data were entered into a Microsoft Excel spread sheet program and then transferred to SPSS Version 20 for analysis. The density of fly population was calculated by dividing the number of flies caught by the number of flies caught by the number of deployed and number of day's deployment and expressed as Flay/Trap/Day [8]. The association between trypanosome infection and risk factors (age, body condition, sex, and PA) was determined by invariable logistic regression. Two sample Student's *t*-tests were used to compare mean PCV of study animals. A statistical significant difference between variables exists when *p* < 0.05 at 95% confidence interval (CI).

## 3. Results

### 3.1. Entomological Result

*Glossina morsitans* and other biting flies such as *Stomoxys*, *Tabanus*, and *Hematopota* were caught. A total of 1512 flies were trapped, and among them, 1162 were tsetse flies while 350 were biting flies, including 135 *Stomoxys*, 115 *Tabanus*, and 100 *Hematopota*. A total of 1162 tsetse flies caught in five PAs were subjected to sexing. 31.6% (367/1162) were males, and 68.4% (795/1162) were females. At all sites in each PA, female tsetse flies were trapped more frequently than males (Table 1).

Out of the total, 1162 (76.8%) were belonging to tsetse of the species *Glossina morsitans submorsitans* followed by *Stomoxys*, 135 (8.6%), *Hematopota*, 100 (6.4%), and *Tabanus*, 115 (7.4%). Only *Glossina morsitans submorsitans* was identified in the survey site with the overall apparent density of 7.7 F/T/D. The highest fly density, 254 (16.7 F/T/D), was observed in the Mender-51 peasant association, and the lowest, 113 (3.8 F/T/D), was recorded in the Mender-47 peasant association in Bambasi District (Table 2).

### 3.2. Parasitological Result

Out of 638 examined cattle, 58 (9.1%) were found to be positive for trypanosome infection using the buffy coat technique. The predominant trypanosomes species were *T*. *congolense* (*n* = 49, 7.7%) followed by *T. vivax* (*n* = 6, 0.9%), *T*. *brucei* (*n* = 1, 0.2%), and mixed infection of *T*. *vivax* and *T. brucei* (*n* = 2, 0.3%). Despite the occurrence of the highest prevalence in Mender-51 (4.5%) and the lowest in Mender-52 PAs (0.5%), a significant difference (*p* > 0.05) was not observed (Table 3).

Concerning age categories, 0.9%, 5.5%, and 2.7% trypanosome infection rates were recorded in young (≤2 years), adult (>2 years; <5 years), and old (≥5 years) cattle, respectively (Table 4). Although as age increased, the prevalence rate also increased. None of these differences were statistically significant. The prevalence of females was less than males. However, statistically no significant difference (*p* > 0.05) was observed between two sex groups.

Of the total 638 sampled animals, 2.8%, 5.5%, and 0.8% prevalence of bovine trypanosomosis was recorded in poor, medium, and good body conditions of the animals, respectively. There was no significant difference (*p* > 0.05) on prevalence of trypanosomosis among animals of different body conditions (Table 4).

### 3.3. Hematological Findings

The mean PCV of anemic (19.60 ± 0.260SE) and normal (29.56 ± 0.228SE) cattle showed significant difference (*p* < 0.05). The proportion of anemic animals infected with parasites was significant (*p* < 0.05) as compared with nonanemic positive animals (Table 5). The risk of being anemic increased by 3.13 (CI = 1.794–5.471) folds when animals were infected with trypanosome (*p* < 0.05).

The overall mean PCV of parasitemic and aparasitemic examined animals was 25.8 ± 0.252SE. The mean PCV value of parasitemic and aparasitemic animals was 23.22 ± 0.989SE and 29.56 ± 0.228SE, respectively. There was a significant difference between the mean PCV value of parasitaemic and aparasitaemic cattle as the mean PCV of parasitic animals was significantly lower than that of aparasitemic animals (*p* < 0.05) (Table 6).

## 4. Discussion

### 4.1. Entomological Study

The entomological survey revealed that *Glossina morsitans submorsitans* is the only species responsible for the cyclical and three genera of biting flies *Stomoxys*, *Tabanus*, and *Hematopota*, which are responsible in mechanical transmission of the trypanosomosis in the area. Overall apparent densities recorded in the current study were7.7 F/T/D and 2.3 F/T/D for *Glossina morsitans submorsitans* and biting flies, respectively. The current findings were in agreement with previous works of [9] at Metekel and Awi zones, Northwest Ethiopia, who reported 6.49 F/T/D tsetse fly, and also with findings of 6 [10] at Bure, Ilubabor Zone, southwestern Ethiopia, who indicated the prevalence of 7.23 and 3.13 F/T/D of tsetse fly and biting fly, respectively.

The result of the current entomological survey is lower than the apparent density of tsetse-flies of 14.97 F/T/D found in selected villages of Arbaminch by [11] and the finding reported by [12] of 19.14 F/T/D apparent density, while they are higher than the values found in the study from Mandura District, Northwest Ethiopia, with 0.06 F/T/D apparent density of tsetse flies [13]. Such wide variations could have resulted from differences in season and density of vegetation cover and types of traps deployed, and type and volume of odour attractants utilized during the studies. The low density of tsetse in the study area may have been due to the expansion of settlements and farmlands in the area. It may also be explained by the migration of the game as a result of climate and habitat changes [14].

The sex ratio of tsetse fly in the current study depicted a higher number of female tsetse species, 795 (68.4%), than male female tsetse species, 367 (31.6%), which is in line with various reports from the country [12, 14]. This finding is also in agreement with [15], as female flies would comprise 70–80% of the mean population. This could be attributed to the longer life span of female compared to male *Glossina* [16].

### 4.2. Parasitological Study

The overall prevalence of cattle trypanosomosis in the study area was found to be 9.1%. The current result agreed with the previous report by [17, 18] who found 11.7% at Jabi Tehran District and 8.57% in western Oromia by [19]. The value found was lower than the report in Gamo-Gofa, 17.33%, by [20] and in Wozeka grid, southern Ethiopia, 27.5%, by [21]. This lower prevalence may occur due to the difference in agroecology of the study area, prophylactic measure, and differences in veterinary services employed in the area which all contribute to the low prevalence of the disease [22].

The overall prevalence of cattle trypanosomosis recorded in current study area was found to be higher than the previous result of [11, 23, 24] and [25] who reported the prevalence of 2.10%, 4.43%, 4.86%, and 4.25% from Didesa District, Arbaminch area, Amhara Region, Northwest Ethiopia, and Ilubabor Zone, southwestern Ethiopia, receptively. The variation between reports might be due to the difference in the management system, season of the study period, the development of drug resistance, and the increase of tsetse challenge due to higher vector density and lack awareness of the animal owners about the disease in the study area.

Three species of trypanosomes were observed in hematological examination, namely, *T. congolense*, *T. vivax*, *T. brucei*, and mixed infection of *T. vivax* and *T. brucei*. This study shows that *T. congolense* was the dominant (84.5%), followed by T. *vivax*, mixed infection of *T. vivax* and *T. brucei*, and *T. brucei* alone. This result was in agreement with [16] who stated that the predominance of *T. congolense* infection in cattle may be also due to the high number of serodems of *T. congolense* as compared to *T. vivax* and the development of better immune response to *T. vivax* by the infected animal. In addition, [26] reported that the dominant trypanosome species in upper Didessa valley of tsetse-infested regions was *T. congolense*. Moreover, [27] stated that the most prevalent trypanosome species in tsetse-infested areas of Ethiopia are *T. congolense* and *T. vivax*.

The prevalence of cattle trypanosomosis was assessed between sexes of animals in the 58 trypanosome positive animals; 25 (3.9%) of them were female animals, and 33(5.2%) of them were male. The trypanosome infection in female animals was similar to male animals as no significant difference was observed (*p* > 0.05); this shows that both male and female cattle are equally susceptible to trypanosomosis infection. This result agrees with the results of [28] and also of [29] who also obtained no significant difference in susceptibility between the two sexes. This also shows equal exposure to the vector of the parasite [30].

As regards the occurrence of the disease in three different body conditions (poor, good, and medium) among the total 638 sampled animals, the highest prevalence was observed in the medium body condition (5.5%) followed by poor (2.8%) and good (0.8%) body conditions, although the difference was not significant (*p* > 0.05). Due to poor body condition, animals are highly susceptible to diseases. This is in line with that emaciated animals are more infected than others, which supports the previous study of [31].

Odds ratio indicated that poor body condition animals are affected eight and five times more than medium and good body condition animals, respectively. This result was in disagreement with [32], who stated that the prevalence of the disease is high in good body condition.

In the current study, highest prevalence of the diseases was 5.5% in adult followed by 2.7% in old and 0.9% in young animals. This could be associated to the fact that animals travel long distance for feed and draught as well as for harvesting crops to tsetse high challenge areas. Young animals are also naturally protected to some extent by maternal antibodies. There was no statistically significant difference between age groups (*p* > 0.05). This result was in agreement with the previous research reported by [33] who stated that there is no statistically significant difference between age groups of the animals.

The mean PCV value of parasitemic animals was found to be 23.22 ± 0.989SE which is significantly lower (*p* < 0.05) than the aparasitemic one which was 29.56 ± 0.252SE. This finding is aligned with previous work by [17, 34]. In the contrary, the number of cattle aparasitemic but anemic was also considerable. The overall incidence of anemia was 36.8% in the research area, and the presence of anemia was higher in trypanosome positive animals (62.5%) than negative animals (34.3%) (Table 5). This may be due to the contribution of trypanosomosis for causing anemia in infected animals. This finding agreed with previous reports by [26] in western Ethiopia in upper Didessa valley. The difference in mean PCV value between parasitemic and aparasitemic animals indicates that trypanosomosis is involved in reducing the PCV values in the infected animals [35].

Among the anemic animals, 31.2% of them were negative to trypanosome infection. This is because the observed anemia can also be caused by other concurrent diseases that can cause anemia such as blood-sucking gastrointestinal parasites, hemiparasite, and malnutrition [36]. However, 3.5% of the cattle with normal PCV value were also found infected by trypanosome and this result is in line with [36] report from East Wollega Zone. This might happen due to the ability of trypanosome positive animal to maintain their PCV value or delayed recovery of the anemic situation after current treatment with trypanocidal drugs. Furthermore, the occurrence of positive animals with normal PCV value might be thought of as recent infections of the animals [37].

## 5. Conclusion and Recommendations

The result of this study 9.1% shows that that the disease is still widespread and infection with trypanosomosis negatively affects the body condition and PCV profile of animals. Thus, it may be responsible for economic losses in Bambasi District of Benishangul-Gumuz Zone which was assumed to be imposed more by the presence of high density of vectors and the situation is getting worse as the prevention and control a of trypanosomosis is facing a challenge due to limitation of vector control activities and chemotherapy. Therefore, it is imperative to extend and strengthen the comprehensive integrated control strategy approach (vector control and chemotherapy) in the studied areas.

## Figures and Tables

**Table 1:** Proportion of the F/T/per male and female *Glossina* species in the study area.

Study site	No. of traps	*Glossina m. submorsitans*			
		Male (%)	Female (%)	Total (%)	F/T/D
Mender-47	15	52 (3.5)	61 (4.1)	113 (7.6)	3.8
Mender-46	15	66 (4.4)	104 (6.9)	170 (11.3)	5.7
Laga Warqee	15	60 (4)	108 (7.2)	168 (11.2)	5.6
Qashimando	15	68 (4.5)	144 (9.6)	212 (14.1)	6.9
Mender-51	15	121 (8.10)	378 (25.2)	499 (33.3)	16.7
Total	75	367 (4.9)	795 (10.6)	1162 (15.5)	7.78

F/T/D = Flay per Trap per Day.

**Table 2:** Fly caught number and their apparent density in different selected areas of the district.

	Tsetse flies caught			Biting flies					
	*Glossina m. submorsitans*			*Stomoxys*		*Tabanus*		*Hematopota*	
Study site	No. of traps	Total number	F/T/D	Total number	F/T/D	Total number	F/T/D	Total number	F/T/D
Mender-47	15	113	3.8	27	0.9	15	0.5	30	1
Mander-46	15	170	5.7	32	1.1	20	0.6	26	0.8
Laga Warqee	15	168	5.6	20	0.6	24	0.8	22	0.7
Qashimando	15	212	6.9	34	1.1	21	0.7	9	0.3
Mender-51	15	499	16.7	22	0.7	35	1.2	13	0.4
Total	75	1162	7.78	135	0.9	115	0.8	100	0.6

F/T/D = Flay per Trap per Day.

**Table 3:** Prevalence of cattle trypanosomosis and identified *Trypanosoma* species in the study area.

Sampling villages	No. of animals examined	No. of +ve cattle	*Trypanosoma* encountered				Prevalence (%)
			*T. con*	*T. vivax*	*T. brucei*	*T*. *vivax* and *T*. *brucei*	
Mender-47	120	7	7	—	—	—	1.1
Mender-46	108	4	3	—	—	1	0.6
Laga Warqee	109	5	5	—	—	—	0.8
Qashimando	118	10	10	—	—	—	1.6
Mender-51	120	29	21	6	1	1	4.5
Mender-52	63	3	3	—	—	—	0.5
Total	638	58	49	6	1	2	9.1

T = trypanosome; +ve = positive.

**Table 4:** Univariable logistic regression analysis of risk factors with the buffy coat result.

Risk factors	Risk factors	No. of animals examined	No. of positive cattle	Prevalence (%)	OR (95% CI)	*p* value
PAs	Mender-47	120	7	1.1%	0.619 (.148–2.593)	0.512
	Mender-46	108	4	0.6	1.240 (.258–5.967)	0.789
	Laga Warqee	109	5	0.8	1.206 (.272–5.353)	0.806
	Qashimando	118	10	1.6	0.680 (.174–2.662)	0.579
	Mender-51	120	29	4.5	0.191 (.052–.699)	0.012
	Mender-52	63	3	0.5	—	—

Sex	Total	638	58	9.1		
	Female	334	25	3.9	1.516 (.836–2.749)	0.171
	Male	294	33	5.2	—	—
	Total	638	58	9.1		

Age	Young (≤2)	108	6	0.9	1.537 (.558–4.234)	0.406
	Adult (>2 ≤ 5)	352	35	5.5	0.895 (.466–1.720)	0.739
	Old (>5)	178	17	2.7	—	—
	Total	638	58	9.1		

BCS	Poor	140	18	2.8	1.086 (.347–3.395)	0.888
	Medium	413	35	5.5	1.332 (.469–3.785)	0.590
	Good	85	5	0.8	—	—
	Total	638	58	9.1		

BCS = body condition score; CI = confidence interval.

**Table 5:** Univariable logistic regression analysis of anemic and nonanemic cattle.

Category	No. of examined animals (%)	Positive (%)	Overall prevalence (%)	Mean ± SE	OR (95% CI)	*p* value
Anemic (<25)	235 (36.8%)	36 (15.3%)	5.6	19.60 ± 0.260	3.13 (1.794–5.471)	*p* ≤ 0.001
Normal (≥25)	403 (63.2%)	22 (5.4%)	3.4	29.56 ± 0.228	—	—
Total	638	58	9.1	25.84 ± 0.252	—	—

**Table 6:** Mean PCV value of parasitemic and aparasitemic animals.

Infection	No. of examined animals	Mean ± SE	OR (95% CI)	*p* value
Parasitemic	58	23.22 ± 0.989	3.13 (1.794–5.471)	*p* ≤ 0.001
Aparasitemic	580	29.56 ± 0.228	—	—
Total	638	25.84 ± 0.252		

## Data Availability

The data used to support the findings of this study are included with the article.
